# Robot-assisted laparoscopic reconstructed management of multiple aneurysms in renal artery primary bifurcations: a case report and literature review

**DOI:** 10.1186/s12894-017-0265-8

**Published:** 2017-10-16

**Authors:** Hai-bin Wei, Xiao-long Qi, Feng Liu, Wang Jie, Xiao-feng Ni, Qi Zhang, En-hui Li, Xuan-yu Chen, Da-hong Zhang

**Affiliations:** 1 Department of Urology, Zhejiang Provincial People’s Hospital, No. 158, Shangtang Road, Xiacheng District, Hangzhou, Zhejiang 310014 China; 2 Department of Nephrology, Sir Run Run Shaw Hospital, No. 3, East Qingchun Road, Jianggan District, Hangzhou, Zhejiang 310076 China; 3 Department of general surgery, Central Hospital of Huzhou, No. 198, Hongqi Road, Wuxing District, Huzhou, Zhejiang 313003 China

**Keywords:** Renal artery aneurysms, Robotic surgery, Reconstruction, Renovascular disease, Case report

## Abstract

**Background:**

Renal artery aneurysm (RAA) is rare and its incidence in the general population remains elusive. There have been few reports on the repair of multiple aneurysms conducted with the Da Vinci robot-assisted surgical platform (Intuitive Surgical Inc., Sunnyvale, CA, USA), especially for those located in renal artery primary bifurcations.

**Case presentation:**

We report our experience in the surgical management of two expanding right-sided RAAs in a 64-year-old man using a robot-assisted laparoscopic approach. Two aneurysms were located in renal artery primary bifurcations, whose diameter was 1.8 and 1.2 cm. The aneurysms were resected and the renal artery branch reconstructed by in situ arteriorrhaphy. The operation lasted for 2 h and 35 min with a warm ischemia time of 26 min and estimated blood loss of 150 ml. The hospital stay was 6 days. The computed tomography (CT) scan performed 2 months after the surgery showed resolution of the aneurysms. Additionally, split renal function indicated the preservation of right renal function in the follow-up period.

**Conclusions:**

The robot-assisted laparoscopic procedure is a safe and effective surgical technique, which may be considered as an alternative to open surgery for complex multiple RAAs in the future.

## Background

Renal artery aneurysm (RAA) is an uncommon clinical condition, whose actual annual incidence rate in the general population remains unclear. The incidence rate of RAAs is estimated to range from 0.03% to 0.09%, according to previous autopsy series reports [1, 2]. There is an increasing detection of RAAs along with the widespread use of ultrasonography and computed tomography (CT) as tools for medical examination and diagnostic purposes. A later research report found an incidence of 0.3% to 2.5% according to some angiographic and CT studies [3]. However, the incidence of multiple RAAs is still uncommon.

The Da Vinci robot-assisted surgical platform (Intuitive Surgical Inc., Sunnyvale, CA, USA) was introduced to our hospital in 2014 and has been used in a variety of minimally invasive urological surgery. It provides possibilities and alternatives for various complicated and delicate surgeries, and has only shown great superiority compared with conventional laparoscopic surgery. Until now, there has been no report on the treatment of multiple RAAs with the Da Vinci robot-assisted surgery. To determine the feasibility of using this approach, we performed a robot-assisted laparoscopic reconstruction of multiple aneurysms at major bifurcations of the renal artery in a 64-year-old man and evaluated the safety and efficiency of the Da Vinci robot-assisted surgery.

## Case presentation

**Fig. 1:**
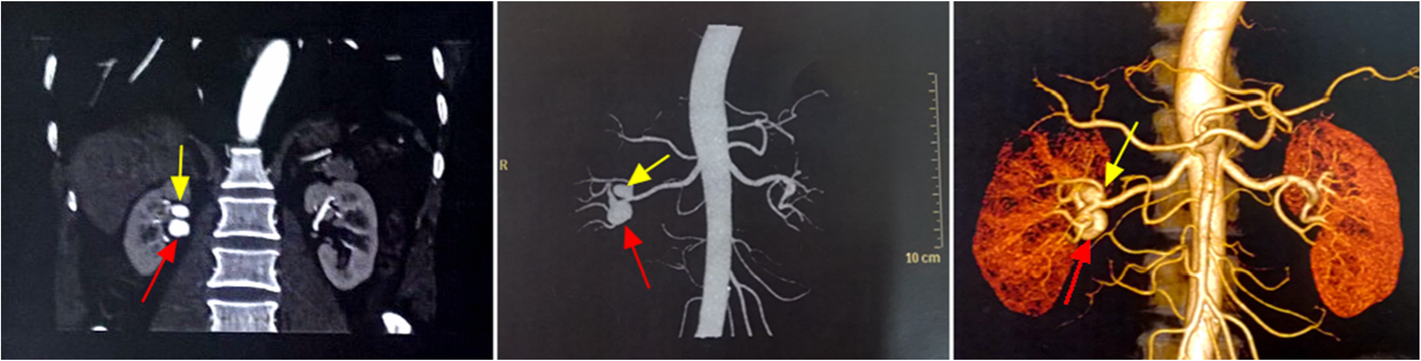
CT angiogram and reconstruction preoperative. Red asterisk indicates the RAA of 1.8 cm. Yellow asterisk represents the RAA of 1.2 cm

A 64-year-old man was admitted to our department due to incidentally discovered RAAs. A previous enhanced CT scan of the local hospital showed two saccular RAAs in the right renal artery. The two aneurysms were located at primary bifurcations of renal artery, with a diameter of 1.8 cm and 1.2 cm, respectively, as shown in Fig. 1. The smaller one was located at the first bifurcation of the first segmental artery of the renal artery, while the other one is at the second bifurcation of the another segmental artery of the renal artery. The patient had no history of comorbidities other than a hepatitis B infection and partial hepatectomy, cholecystectomy and splenectomy due to hepatic carcinoma in 2002. Also, there were no typical clinical symptoms, such as flank pain, hematuria, or a sense of abdominal distension. His blood pressure was 106/56 mmHg at admission and the usual hypertension was absent.

Split renal function was evaluated through glomerular filtration rate (GFR) before surgery and then 2 months after surgery. We obtained the GFR using the camera-based Gate’s method to measure the renal uptake of ^99m^Technetium (^99m^Tc) diethylenetriaminepentaacetic acid (DTPA). The preoperatively estimated GFRs were 30.16 ml/min/1.69 m^2^ (right renal) and 36.09 ml/min/1.69 m^2^ (left renal). The class 2 classification was deemed according to the American Society of Anesthesiologists (ASA).

The patient was secured in the left lateral position. A Veress needle was used to establish a carbon dioxide (CO_2_) pneumoperitoneum of 12 mmHg lateral to the right rectus muscle at the level of the umbilicus, and then a 12-mm laparoscopic camera port was placed at the same position. A 30° lens was introduced to position the other ports under direct vision. A 12-mm port was placed 3 cm below the umbilicus position. Then, two ports of 8-mm were placed at 10 cm below the xiphoid at the ventral midline and below the 12th rib costochondral margin at the mid-clavicle. The 5-mm assistant port was inserted at 2 cm below the xiphoid at the ventral midline.

**Fig. 2:**
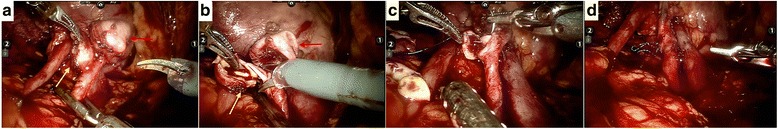
The surgical procedure of reconstruction. **a**, RAA dissected and renal hilar control. **b**, resection of RAA. **c**, reconstruction of RAA. **d**, completed reconstruction of renal artery main trunk. Red asterisk indicates the RAA of 1.8 cm. Yellow asterisk represents the RAA of 1.2 cm

The renal hilum was completely dissected anteriorly and posteriorly, and the right renal artery and segmental arteries were isolated at the level of the renal hilum. The two RAAs were fully exposed and turned out to be saccular in accordance with the preoperative CT scan. The vascular bulldog clamp was used to clamp the inflow of renal artery. Then, the RAAs were resected from the proximal inflow to the distal outflow by cold monopolar shears, preserving the vascular back wall intact for the ensuing repair process. The bifurcations were then reconstructed with continuous suture using 5–0 Prolene (Ethicon US, LLC., Somerville, NJ, USA). Before the last suture, the vascular bulldog clamp was removed and then blood filled the entire renal artery to exclude intravascular air. Subsequently, intraoperative ultrasound was adopted for arterial perfusion and hemodynamic evaluation. The surgical procedure is depicted in Fig. 2. Finally, the Da Vinci device was undocked after removal of the resected RAAs by the Endocatch bag.

**Fig. 3:**
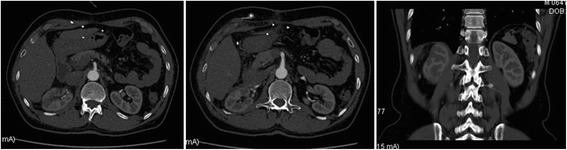
CT angiogram postoperative

The warm ischemia time was 26 min. The total operative duration and estimated blood loss was 155 min and 150 ml, respectively. There was no perioperative complication. The serum creatinine and blood urea nitrogen was stable within the reference range. On postoperative day 6 the patient was discharged home. The CT scan performed 2 months later, shown in Fig. 3, demonstrated resolution of the aneurysms without any recurrence or artery stenosis; the estimated GFR were 29.76 ml/min/1.69 m^2^ (right renal) and 34.03 ml/min/1.69 m^2^ (left renal).

## Discussion and conclusions

RAA is a rare disorder, and multiple RAAs, which occur in about 18% of the RAA cases, are even more rare in the general population [4]. It is difficult to reach a consensus on the appropriate indications for intervention in RAAs, due to the numerous aspects involved, such as the clinical symptoms (hematuria, refractory hypertension, persistent back pain and renal infarction), the anatomical and morphological characteristics of RAAs (size, location, wall calcification and enlarging lesion), and general clinical features (life expectancy, comorbidities and planned pregnancy) [5]. Currently, widely accepted indications for RAA include more than 2 cm in size, women in reproductive age planning for pregnancy and positive clinical manifestation, such as pain, hematuria and refractory hypertension attributable to the RAA. In our opinion, the coexistence of two RAAs in the renal artery added to the risk of rupture and progression, which also required active intervention.

RAAs can be managed by surgical or percutaneous interventional radiological treatments. Due to lower invasiveness and reduced morbidity, percutaneous interventional radiological techniques surpass traditional surgical techniques and have become increasingly popular [5]. However, endovascular techniques are not preferred for all conditions, such as aneurysms located at the artery bifurcations or distal branches. As the gold standard for RAAs, open surgery results in the most considerable blood loss, prolonged recovery, and the heaviest patient burden [6, 7]. Since a report on laparoscopic repair of RAAs by Gill et al., in 2001, more and more successful laparoscopic repairs have been reported [8].

The laparoscopic technique has not been widely accepted because laparoscopic surgical reconstruction of RAA is still a challenging and time-consuming task under the constraint of the warm ischemia time (WIT), especially for those involving multiple aneurysms. First, intracorporeal suturing for angioplasty remains technically difficult, especially for aneurysms located at artery bifurcations, as well as those located at distal branches. Second, intracorporeal angioplasty is a time-consuming step after the renal artery is clamped with the bulldog clamp. It is generally considered that optimal ischemia time should not exceed 35 min [9]. In addition, there are considerable perioperative complications, such as unplanned nephrectomy and artery reintervention resulting from anastomotic stenosis or thrombosis [6]. The Da Vinci surgical platform can provide precise suturing thanks to the perfect features of the “wristed” manipulators and 3-D stereo imaging. Accordingly, robot-assisted laparoscopic surgery may overcome some of the challenges of suturing and angioplasty, and offers an alternative surgical approach for the treatment of RAAs.

One of the key limiting factors for surgery is the WIT. Every minute of WIT matters when the renal artery is clamped, since an additional minute of WIT is associated with a 6% increase in the risk of new-onset stage IV chronic kidney disease during the follow-up [10]. Two artery aneurysms were resected and reconstructed with a WIT of 26 min, which was in a controllable range. There are several main reasons for a shorter WIT. First, the flexible “wristed” instruments and 3-D optical imaging of the Da Vinci surgical platform make it easier to cut and suture for reconstruction. Second, arteriorrhaphy greatly reduced the difficulty of angioplasty and avoided more artery suturing due to vascular anastomosis and aneurysmectomy with bypass. Third, the vascular bulldog clap was removed before the last suture. Such early withdrawal of the bulldog clap further reduced the WIT and intravascular air was excluded by the filling blood. Also, since the two aneurysms were close in the renal artery, the reconstruction of the two aneurysms was completed after only one clamping of the renal artery, which avoided acute kidney injury caused by repeatedly occluding the renal artery. Due to a WIT of less than 30 min, the cooled (4 °C) renal perfusion supplemented with mannitol or prostaglandin E was avoided. Thus, despite having performed two reconstructions in the renal artery, the split renal function was not affected, as shown on the ^99m^Tc-DTPA renogram performed 2 months later.

We systematically searched the Medline electronic database, and summarized the case series on robot-assisted laparoscopic RAA reconstruction. To the best of our knowledge, there are only a few reports on the Da Vinci robot-assisted reconstruction of RAA [7, 11, 12], as listed in Table 1. Like our study, the study by Luke as well as that by Samarasekera, are all single case reports, and there were no severe complications requiring acute reintervention [7, 11]. In the study by Giulianotti, one out of 5 patients had elevated postoperative serum creatinine level, which spontaneously returned to normal range by postoperative day three [12]. In addition, another patient experienced stenosis in the reconstructed branch 6 months after the repair, and responded well to percutaneous angioplasty [12]. Although we performed arteriorrhaphy for two RAAs at the same time, the complications did not increase compared with the other three studies. In general, the Da Vinci robot-assisted reconstruction of RAA is a safe technique associated with a low complication rate, despite the adoption of different angioplasty ways to reconstruct.

**Table 1:** Summary of case series of robot-assisted laparoscopic RAA reconstruction

	Our study-2015	Luke-2006	Giulianotti-2010	Samarasekera-2014
Patient number	1	1	5	1
Robot	DaVinci Si	DaVinci	DaVinci	DaVinci Si
Size (cm)	1.8 and 1.2	2.5	1.94 (range, 9–28)	1.6
Suture type	Arteriorrhaphy	End-to-end anastomosis	1 end-to-end anastomosis and 4 graft angioplasty	Arteriorrhaphy
Perioperative characteristic				
WIT (min)	26	59	10 and 38.5 (range, 20–60)	44
Operative time (min)	155	360	288 (range, 170–360)	240
Blood loss (ml)	150	650	100 (range, 50–300)	260
Hospital stay (d)	6	3	5.6 (range, 3–7).	-
Follow-up (months)	10	-	28 (range, 6–48)	2
Complications				
Perirenal hematoma	0	0	0	0
Haemorrhage	0	0	0	0
Renal dysfunction	0	0	1	0
Unimproved hypertension	0	0	3	0
Stenosis	0	0	1 patient treat with percutaneous angioplasty	0

*RAA* renal artery aneurysm, *WIT* warm ischemia time

In summary, the results of our study demonstrate the safety and efficiency of the robot-assisted laparoscopic technique to treat complex multiple RAAs. Although more technical refinements and longer follow-up period is necessary, the robot-assisted laparoscopic technique represents a valid alternative to open surgery for complex multiple RAAs in the future.

## Abbreviations

ASA: American Society of Anesthesiologists
CO_2_: Carbon dioxide
CT: Computed tomography
GFR: Glomerular filtration rate
RAA: Renal artery aneurysm
WIT: Warm ischemia time
