# Correction to: Robot-assisted laparoscopic reconstructed management of multiple aneurysms in renal artery primary bifurcations: a case report and literature review

**DOI:** 10.1186/s12894-017-0291-6

**Published:** 2017-11-03

**Authors:** Hai-bin Wei, Xiao-long Qi, Feng Liu, Jie Wang, Xiao-feng Ni, Qi Zhang, En-hui Li, Xuan-yu Chen, Da-hong Zhang

**Affiliations:** 1 Department of Urology, Zhejiang Provincial People’s Hospital, No. 158, Shangtang Road, Xiacheng District, Hangzhou, Zhejiang 310014 China; 2 Department of Nephrology, Sir Run Run Shaw Hospital, No. 3, East Qingchun Road, Jianggan District, Hangzhou, Zhejiang 310076 China; 3 Department of general surgery, Central Hospital of Huzhou, No. 198, Hongqi Road, Wuxing District, Huzhou, Zhejiang, 313003 China

## Correction

After publication of this work [1] it was noticed the author – Jie Wang’s name was in the wrong order.

The original article was corrected.

The publisher apologises for this error.
